# HCV Genomic RNA Activates the NLRP3 Inflammasome in Human Myeloid Cells

**DOI:** 10.1371/journal.pone.0084953

**Published:** 2014-01-06

**Authors:** Wei Chen, Yongfen Xu, Hua Li, Wanyin Tao, Yu Xiang, Bing Huang, Junqi Niu, Jin Zhong, Guangxun Meng

**Affiliations:** 1 Key Laboratory of Molecular Virology & Immunology, Institut Pasteur of Shanghai, Chinese Academy of Sciences, Shanghai, China; 2 Department of Hepatology, The First Hospital of Jilin University, Changchun, China; University of Iowa Carver College of Medicine, United States of America

## Abstract

**Background:**

Elevated plasma levels of IL-1β and IL-18 from patients with hepatitis C virus (HCV) infection indicate a possible activation of inflammasome by HCV.

**Methodology/Principal Findings:**

To demonstrate whether HCV infection activates the inflammasome, we investigated inflammasome activation from HCV infected hepatic Huh7 cells, or monocytic cells and THP-1 derived macrophages challenged with HCV virions, but no any inflammasome activation was detected in these cells. However, when we transfected HCV genomic RNA into monocytes or macrophages, IL-1β was secreted in a dose-dependent manner. We also detected ASC oligomerization and caspase-1 cleavage in HCV RNA transfected macrophages. Using shRNA-mediated gene silencing or specific inhibitors, we found that HCV RNA-induced IL-1β secretion was dependent on the presence of inflammasome components such as NLRP3, ASC and caspase-1. Furthermore, we also found that RIG-I was dispensable for HCV RNA-induced NLRP3 inflammasome activation, while reactive oxygen species (ROS) production was required.

**Conclusions:**

Our results indicate that HCV RNA activates the NLRP3 inflammasome in a ROS-dependent manner, and RIG-I is not required for this process.

## Introduction

Hepatitis C virus (HCV) infection tends to become persistent and causes liver fibrosis and cirrhosis due to chronic inflammation in humans [1]. The 9.6-kb genome of HCV ssRNA is composed of a 5′ untranslated region (5′UTR), a single open reading frame (ORF) and a 3′UTR, as well as an internal ribosome entry site (IRES) within the 5′UTR, which directs translation of a polyprotein precursor of about 3000 amino acids that is cleaved into mature structural and non-structural proteins [2], [3]. It was reported that the HCV 5′-ppp poly-U/UC RNA variants stimulate strong retinoic acid-inducible gene I (RIG-I) activation *in vitro* [4]. RIG-I was also reported to detect *in vitro* transcribed HCV RNA, RNA without a 5′-triphosphate end, 5′-triphosphate single-stranded RNA and short double-stranded RNA for type I interferon production [5]–[7].

Besides the anti-viral type I interferon response, pro-inflammatory cytokines such as tumor necrosis factor (TNF)-α and interleukin (IL)-6 can also be induced upon HCV infection [8]–[10]. Recently, serum IL-18 and IL-1β levels have been observed to be clearly higher in patients with chronic HCV infection and HCV related cirrhosis than in healthy controls, and IL-18 was taken as marker of the acute phase of HCV infection [8], [11]–[15]. As a special group of cytokines, the secretion of IL-1β and IL-18 involves a two step process: step 1 is the synthesis of pro-IL-1β and pro-IL-18 (signal 1); step 2 is activation of caspase-1 (signal 2) which cleaves pro-IL-1β and pro-IL-18 into mature IL-1β and IL-18 [16]–[18]. Recently it was found that the activation of caspase-1 is mediated by the inflammasome, a protein complex composed of PRRs including AIM2 (Absent In Melanoma 2) or NLRP3 (NOD-like receptor family, pyrin domain containing 3), adaptor protein ASC (apoptosis-associated specklike protein containing a CARD) as well as pro-caspase-1 [16], [19]. Other reported inflammasomes include NLRP1-, NLRC4-, NLRP6-, NLRP7- as well as RIG-I-inflammasome [20]–[22]. Various microbes are able to activate inflammasomes [23], and the NLRP3 and RIG-I inflammasomes were reported to be activated by RNA viruses [24]–[27]. Thus, elevated IL-1β and IL-18 levels in HCV-infected patients indicate that HCV may trigger inflammasome activation.

Recently, Burdette *et.al.* reported that HCV (JFH-1) infection induced NLRP3 inflammasome activation in the hepatoma cell line Huh7.5 [28]. However, the expression of inflammasome components was found to be prominent in Kupffer cells (KC) and liver sinusoidal endothelial cells, moderate in periportal myofibroblasts and hepatic stellate cells, virtually absent in primary hepatocytes [29], therefore, inflammasome activation in hepatocytes may not be the main origin of IL-β from HCV infected patients. Instead, HCV virions or its components such as genomic RNA may activate the inflammasome in KC or peripheral myeloid cells, and this should be the main origin of IL-β. Interestingly, a more recent study from Negash *et al.* revealed that there was no appreciable IL-1β from HCV infected hepatoma cells or primary hepatocytes, while robust IL-1β production was induced by HCV virions in human macrophages [30].

In our present study, no inflammasome activation was observed in HCV infected Huh7 or Huh7.5.1 cells. Moreover, we found that HCV virions did not trigger IL-1β secretion in human myeloid cells. However, we discovered that HCV RNA transfection in monocytes or macrophages induced robust IL-1β secretion, which was dependent on the NLRP3 inflammasome. HCV RNA transfection triggered ASC oligomerization and caspase-1 cleavage, suggesting that the HCV genome possesses the ability to activate signal 2 directly. In addition, we found that neither IL-1β secretion nor caspase-1 cleavage was dependent on RIG-I.

## Materials and Methods

### Primary Monocyte Isolation and Cell Culture

Human PBMCs were obtained from the Shanghai Blood Center (Shanghai, China). Human monocytes were separated by Percoll™ density-gradient centrifugation (G.E Healthcare, Bio-sciences, Sweden) from isolated PBMCs. Monocyte derived macrophages (MDM) were generated by incubation of primary monocytes with recombinant M-CSF (20 ng/ml) for a week as described [30]. THP-1 cells were maintained in RPMI 1640 media, supplemented with 10% FBS, 100 IU/ml penicillin, 1 mg/ml streptomycin, 0.25 µg/ml amphotericin B, non essential amino acids, 1 mM sodium pyruvate, 10 mM HEPES buffer and 2 mM glutamine. THP-1 cells were differentiated to macrophage-like cells with 100 nM phorbol-12-myristate-13-acetate (PMA) for 3 hours and then rested for 48 hours before experiments. In some indicated experiments, THP-1 cells were differentiated to macrophages by treatment with 40 nM of PMA overnight at 37°C as described by Negash et al [30].

### HCVcc Preparation, Purification and HCV RNA Generation

The methods of HCVcc preparation had been described [31]. Harvested HCVcc was purified by sucrose density gradient centrifugation and titrated [31]. To generate the full-length genomic RNA, the 1–807 bp, 2406–3256 bp, 5626–6437 bp and 3′UTR of the HCV JFH-1 strain [32] and the pJFH-1 plasmids containing T7 promoter were linearized at the 3′ of the HCV cDNA by XbaI digestion [33], which was used as the template for *in vitro* transcription (Ambion, Austin, TX, USA).

### Quantification of IL-1β Secretion by ELISA

Supernatants were analyzed for cytokine IL-1β secretion by ELISA (BD Biosciences, San Diego, CA) according to the manufacturer’s instructions.

### Quantitative Real-time PCR

RNA from human monocytes or Huh7 cells were extracted using RNA Lyzol reagent (EXcell Bio, China). cDNA was synthesized with the Rever TraAce®qPCR RT Kit (TOYOBO.CO, TLD, Japan). Quantitative real-time PCR was performed on a 7900 Fast Real-Time PCR System (AB Applied Biosystems, USA) using SYBR® Green Realtime PCR Master Mix (TOYOBO.CO, TLD, Japan). The specificity of amplification was assessed for each sample by melting curve analysis. Relative quantification was performed using standard curve analysis. The quantification data are presented as a ratio to the control level. The *Homo sapiens* (hs) gene specific primers used were as follows:

IFN-β, 5′-GATTCATCTAGCACTGGCTGG-3′ (forward) and 5′- CTTCAGGTAATGCAGAATCC-3′ (reverse);

RIG-I, 5′-CCTACCTACATCCTGAGCTACAT-3′ (forward) and 5′-TCTAGGGCATCCAAAAAGCCA-3′ (reverse);

IL-1β, 5′-CACGATGCACCTGTACGATCA-3′ (forward) and 5′-GTTGCTCCATATCCTGTCCCT-3′ (reverse);

ASC, 5′-AACCCAAGCAAGATGCGGAAG-3′ (forward) and 5′-TTAGGGCCTGGAGGAGCAAG-3′ (reverse);

Actin, 5′-AGTGTGACGTGGACATCCGCAAAG-3′ (forward) and 5′-ATCCACATCTGCTGGAAGGTGGAC-3′ (reverse);

NLRP3, 5′-AAGGGCCATGGACTATTTCC-3′ (forward) and 5′-GACTCCACCCGATGACAGTT-3′ (reverse);

Caspase-1, 5′-TCCAATAATGCAAGTCAAGCC-3′ (forward) and 5′-GCTGTACCCCAGATTTTGTAGCA-3′ (reverse).

### RNA Transfection into Myeloid Cells

RNA including negative control tRNA, positive control Poly I:C, HCV 1–807 bp, 2406–3256 bp, 5626–6437 bp, HCV 3′UTR, HCV full length (FL) RNA, ssRNA40, ssRNA41 and ssPolyU (Invivogen, USA) were transfected with Lipofectamine 2000 (Invitrogen, USA) diluted in OptiMEM (Invitrogen, USA) without nucleic acids according to the manufacturer’s protocol. 1 µg of nucleic acid were delivered into THP-1 cells or THP-1 derived macrophages with 2.5 µl of Lipofectamine 2000 unless described otherwise.

### Generation of THP-1 Cells Expressing shRNAs Targeting Genes of Interest

Three human RIG-I coding sequences were selected for construction of specific shRNA: RIG-I-1, ntGTGGAATGCCTTCTCAGAT; RIG-I-2, nt GCTTCTCTTGATGCGTCAGTGATAGCAAC; RIG-I-3, nt GATAGAGGAATGCCATTACACTGTGCTTG. Of them, shRNA RIG-I-3 silenced cells were applied for function experiments. Similarly, three human AIM2 coding sequences were selected for construction of specific shRNA: AIM2-1, nt GCCTGAACAGAAACAGATG; AIM2-2, nt ATACAAGGAGATACTCTTGCTAACAGGCC; AIM2-3 nt CCCGAAGATCAACACGCTTCA. In this case, shRNA AIM2-1 silenced cells were applied for function experiments. shRNA vectors against human NLRP3, caspase-1, ASC, and their scramble vectors are gifts from Dr. Jurg Tschopp [34]. Briefly, THP-1 cells stably expressing shRNA were obtained as follows: ntGATGCGGAAGCTCTTCAGTTTCA of the human ASC coding sequence, ntCAGGTACTATCTGTTCT of the human NLRP3 coding sequence, ntGTGAAGAGATCCTTCTGTA of the 3′UTR of the human caspase-1 were inserted into pSUPER. The Pol III promoter shRNA cassettes from these vectors and from a lamin A/C-specific pSUPER control construct were inserted into the lentiviral vector pAB286.1, a derivative of pHR that contains a SV40-puromycin acetyl transferase cassette for antibiotic selection. Second-generation packaging plasmids pMD2-VSVG and pCMV-R8.91 [35] were used for lentivirus production.

### Immunoblotting

For immunoblotting, cells were lysed with buffer (10 mM Tris pH 7.5, 1% NP-40, 150 mM NaCl, and protease inhibitor cocktail). Proteins were separated on sodium dodecyl sulphate-polyacrylamide gels and then transferred onto polyvinylidene difluoride membranes. The membranes were blocked with 5% milk in 1 X TBS with 0.5% Tween-20 and then probed with primary antibodies as follows: rabbit anti-human mature (17 kDa) IL-1β (D116, Cell Signaling, USA), goat anti-human pro-IL-1β (31 kDa) (sc-1250, Santa Cruz, USA), rabbit anti-human caspase-1 (sc-515, Santa Cruz, USA), and monoclonal mouse anti-human β-actin (KM9001, Tianjin Sungene Biotech, China). Appropriate HRP-conjugated secondary antibodies were used and signals were detected using ECL reagent (Amersham, USA).

### Statistical Analysis

Data were analyzed for statistical significance by the two-tailed student’s t test and values were shown as mean ± standard deviation (SD) if not described otherwise. Differences in P values ≤0.05 were considered as statistically significant.

## Results

### HCV Infection does not Induce IL-1β Secretion in Huh7 Cells

**Figure 1:**
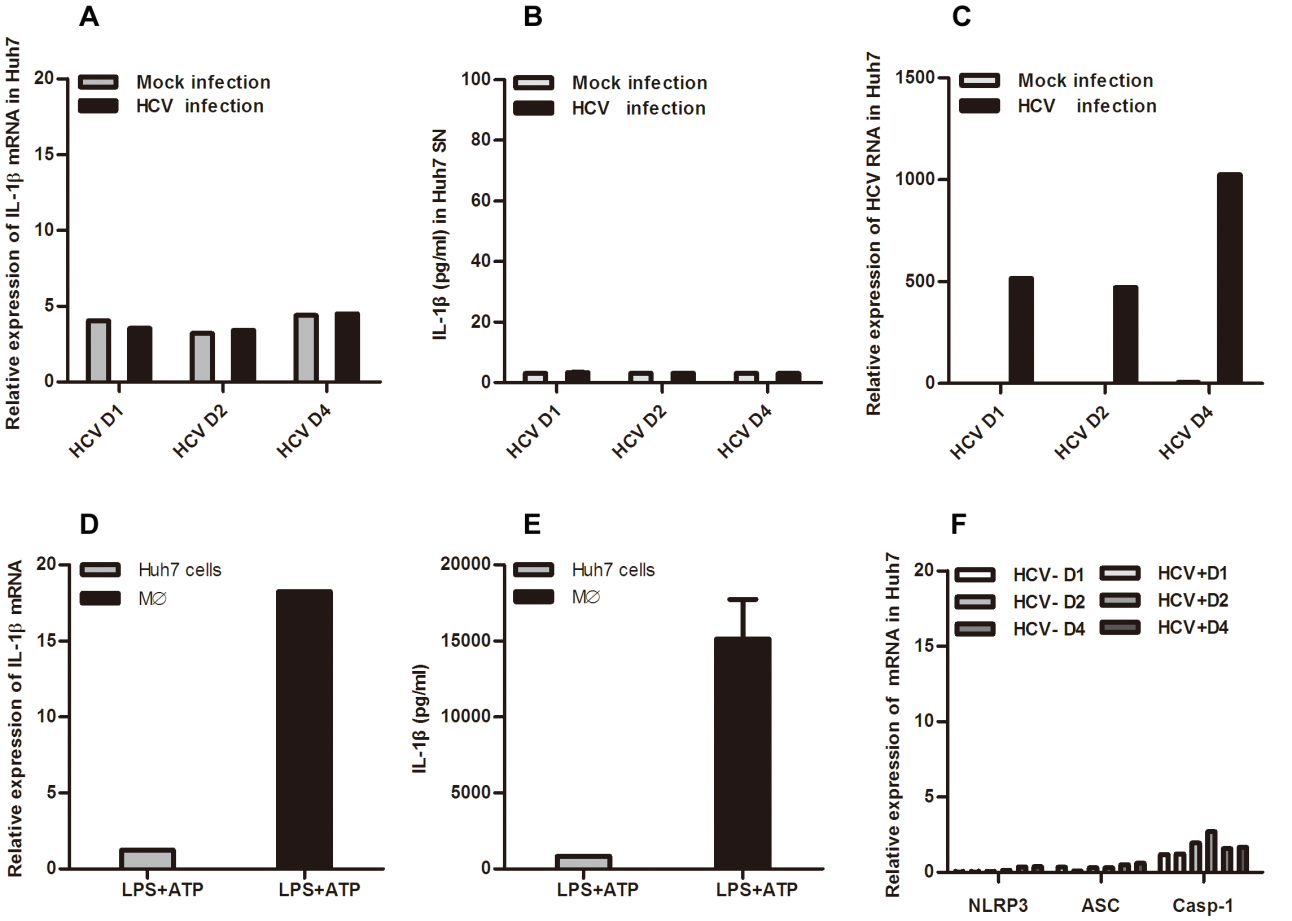
HCV infection does not induce IL-1β secretion in Huh7 cells. Huh7 cells were incubated with HCV virions (MOI = 1) for 1, 2 or 4 days. Total RNA was extracted for Q-PCR analysis (A, C, F) and supernatants were harvested for IL-1β ELISA testing (B). THP-1 derived macrophages and Huh7 cells were incubated with LPS (200 ng/ml for 6 hours) followed by ATP pulsing (5 mM) for 30 minutes, the cells were then collected for IL-1β mRNA detection by Q-PCR (D), and supernatants were harvested for IL-1β ELISA (E). Data shown here represent at least three independent experiments performed with internal triplicates.

To demonstrate the possible production of IL-1β from HCV-infected hepatoma cells, cellular lysates and the supernatants (SNs) from HCV virion-incubated Huh7 cells were collected at indicated time points for analysis (Figure 1A–C). We found that the level of IL-1β mRNA was not elevated in HCV (JFH-1) infected Huh7 cells (Figure 1A), nor was the IL-1β protein being detected in SNs from these cells at day 1, day 2 or day 4 after virus infection (Figure 1B), although the infection efficiency was found normal as indicated by HCV RNA replication (Figure 1C). Moreover, in another hepatoma cell line Huh7.5.1 cells, 4 days after HCV infection, no IL-1β was detected either (Figure S1). To examine the potential low level activation of the inflammasome in Huh7 cells, we treated the cells with LPS and ATP, but IL-1β production was still not detected (Figure 1D–E). We next detected the expression levels of the inflammasome components in HCV JFH-1-infected Huh7 cells, and found that there was nearly no inflammasome components expressed (Figure 1F), which was similar to a previous report [29]. Therefore, we did not detect any IL-1β secretion in HCV infected hepatoma cell lines.

### HCV Particles do not Induce IL-1β Secretion from Human Monocytes and Macrophages

**Figure 2:**
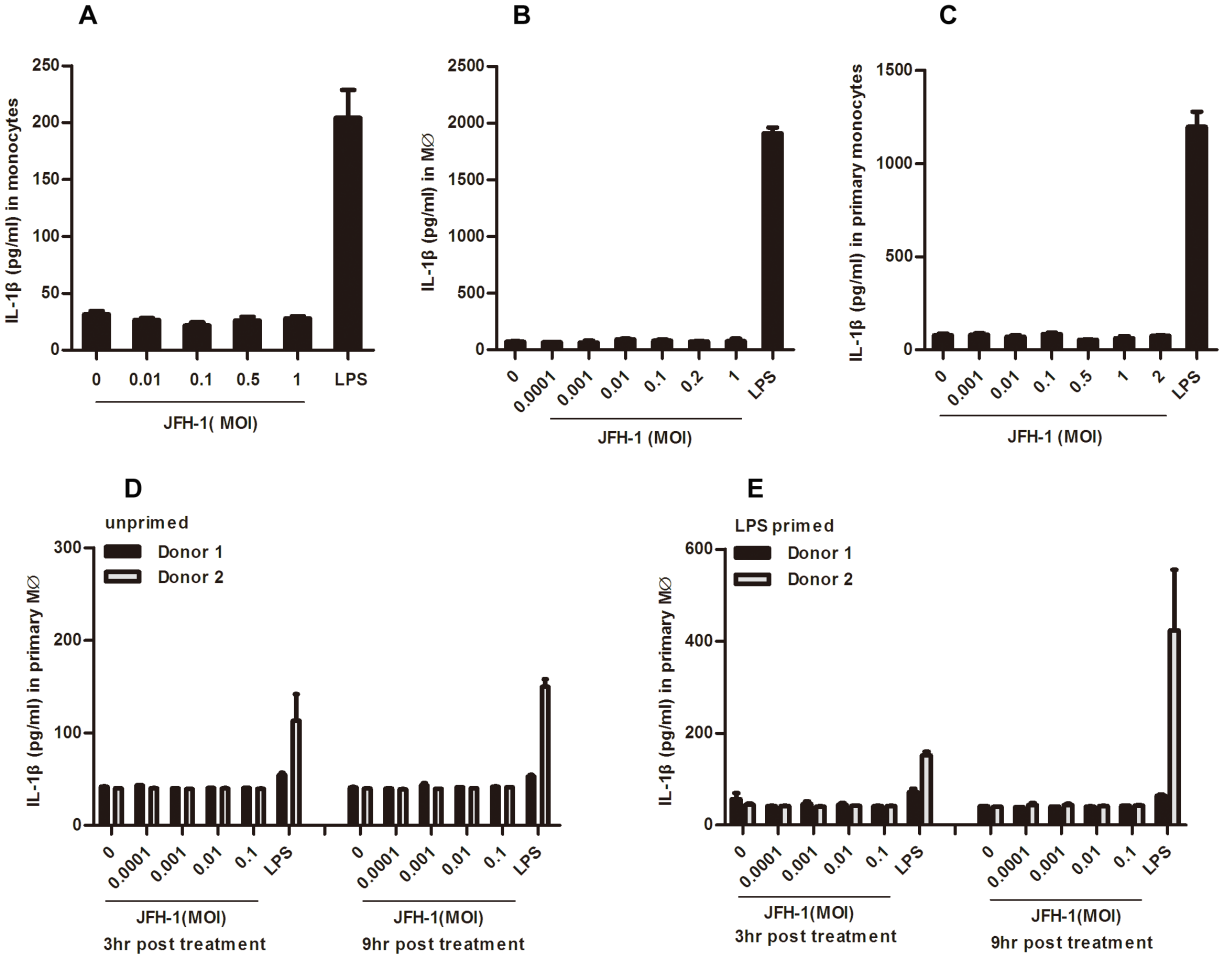
HCV virion treatment does not trigger IL-1β secretion in human myeloid cells. THP-1 cells (A), THP-1 derived macrophages (B), human primary monocytes (C), human primary unprimed (D) and LPS primed (E) macrophages were treated with purified HCV virions at different MOI for 12 hours and the supernatants were harvested for IL-1β ELISA testing. Data shown here represent the mean ± SD of at least three independent experiments performed with internal triplicates.

Since clinical reports have shown that IL-1β and IL-18 were up-regulated in HCV infected patients [8], [11]–[15] and there exists abundant expression of inflammasome components in monocytes and macrophages [17], we speculated that HCV virion and/or its components may activate the inflammasome in myeloid cells. However, when we treated THP-1 monocytes (Figure 2A), THP-1 derived macrophages (Figure 2B), human primary monocytes (Figure 2C) and macrophages (either unprimed or LPS primed) (Figure 2D–E) with purified HCV virions at a multiplicity of infection (MOI) from 0.001 to 2 as indicated, no any IL-1β secretion was detected. Therefore, our results indicated that the phagocytosis of HCV by monocytes or macrophages may not be sufficient to activate the inflammasome. However, Negash *et al.* found that HCV virions induced robust IL-1β secretion from macrophages [30]. We speculated that the THP-1 differentiation procedures between Negash’s and ours were different. However, when we applied the exact same differentiation procedure, we still could not detect any IL-1β in HCV treated macrophages (Figure S2). Perhaps other differences in cell culture condition accounted for the different observation.

### HCV RNA Induces IL-1β Secretion in Macrophages

**Figure 3:**
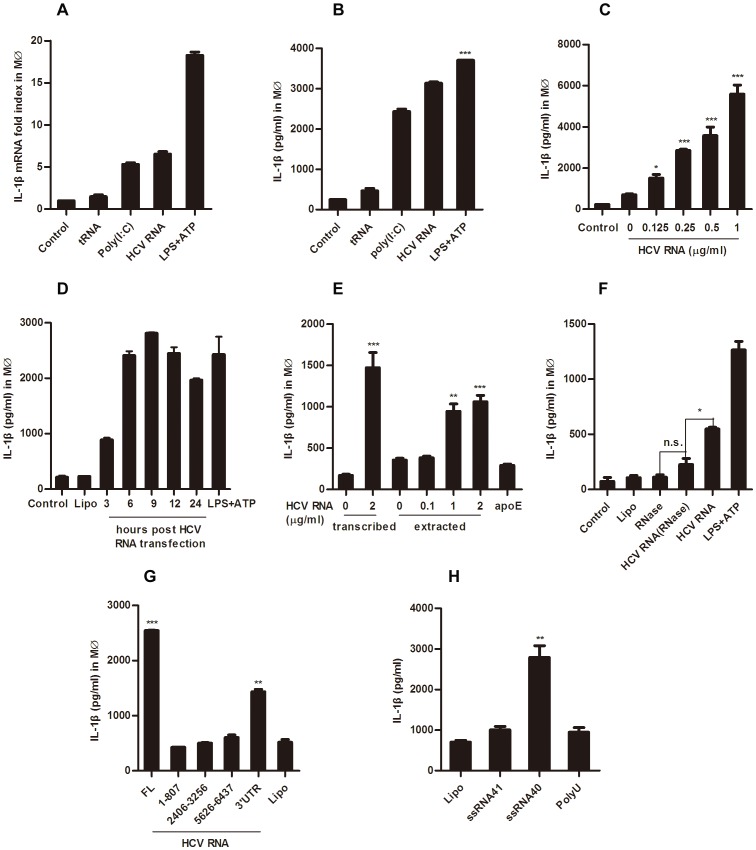
HCV RNA induces IL-1β production in macrophages. THP-1 derived macrophages were stimulated with 2 µg/ml of yeast tRNA, poly (I:C) and HCV genomic RNA for 6 hours, cells and supernatants were collected for IL-1β mRNA and protein detection by Q-PCR and ELISA, respectively (A, B). Macrophages were stimulated with different doses of HCV RNA for 6 hours (C), or with 2 µg/ml HCV RNA for different time periods (D), and then the supernatants were harvested for IL-1β ELISA. E, Macrophages were stimulated for 6 hours with different doses of *in vitro* transcribed HCV RNA and HCV RNA extracted from purified HCV virions through a sucrose cushion, and the supernatants were harvested for IL-1β ELISA; ApoE served as a negative control and LPS+ATP was set as a positive control. HCV RNA digested with RNase (F), different motifs of HCV RNA (G) and ssRNA40, ssRNA41, polyU (H) were transfected into THP-1 derived macrophages, 6 hours later the supernatants were harvested for IL-1β ELISA. Data presented are mean ± SD of one representative of three independent experiments. B, ***represents P<0.001, **represents P<0.01 and *represents P<0.05 in comparison with control during statistical analysis.

Although we found that HCV virions did not activate the inflammasome in hepatoma cell lines or myeloid cells, we believe that some components instead of the HCV virion particle itself could activate the inflammasome, because several reports showed high plasma levels of IL-18 and IL-1β in HCV infected patients [8], [11]–[15]. Since HCV RNA is a well known PAMP *in vivo* and *in vitro* [4], [32], [36], we evaluated the ability of HCV RNA in triggering inflammasome activation in THP-1 derived macrophages. We transfected HCV RNA obtained from *in vitro* transcription into macrophages, followed with IL-1β assay. In this experiment, clear IL-1β mRNA up-regulation and IL-1β protein secretion was observed (Figure 3A–B). In addition, HCV RNA induced IL-1β production in a dose dependent manner (Figure 3C). In a time kinetics test, IL-1β secretion was increased from 3 h to 6 h post HCV RNA transfection and remained at a steady level till 24 h after transfection (Figure 3D). Moreover, genomic RNA extracted from purified HCV virions exhibited similar induction of IL-1β (Figure 3E). To exclude the possibility of contamination in the RNA preparation, we applied the unrelated ApoE transcript as a control, which led to only background level of IL-1β secretion compared with HCV RNA (Figure 3E). To further exclude the possibility that some contamination might have caused IL-1β induction, we digested the HCV RNA with RNase. The result showed that it was the HCV RNA itself that accounted for the IL-1β induction from myeloid cells, as RNase treated HCV RNA lost the ability to induce IL-1β release (Figure 3F).

Moreover, we went a step further to demonstrate which part of the HCV genome might have been accounting for the IL-1β induction in macrophages. When different fragments of the HCV genomic RNA was transfected under the same molar concentration (0.3 pM), we found that only the 3′UTR contained the crucial motif for IL-1β induction, although it was not as potent as the full-length HCV genomic RNA (Figure 3G). It had been reported that transfection with EMCV RNA fails to stimulate IL-1β secretion [37], while uridine-rich single-stranded RNA40 (ssRNA40) from the HIV-1 long terminal repeat is able to induce IL-1β production [26]. Our study and others also confirmed that ssRNA40 but not ssRNA41 nor Poly U was able to induce IL-1β secretion (Figure 3H) [38]. These data suggest that not all virus RNA is able to activate macrophages and certain specific sequence or structure is critical for HCV RNA-induced IL-1β secretion.

### HCV RNA Transfection Activates the Inflammasome Through NLRP3 but not RIG-I

**Figure 4:**
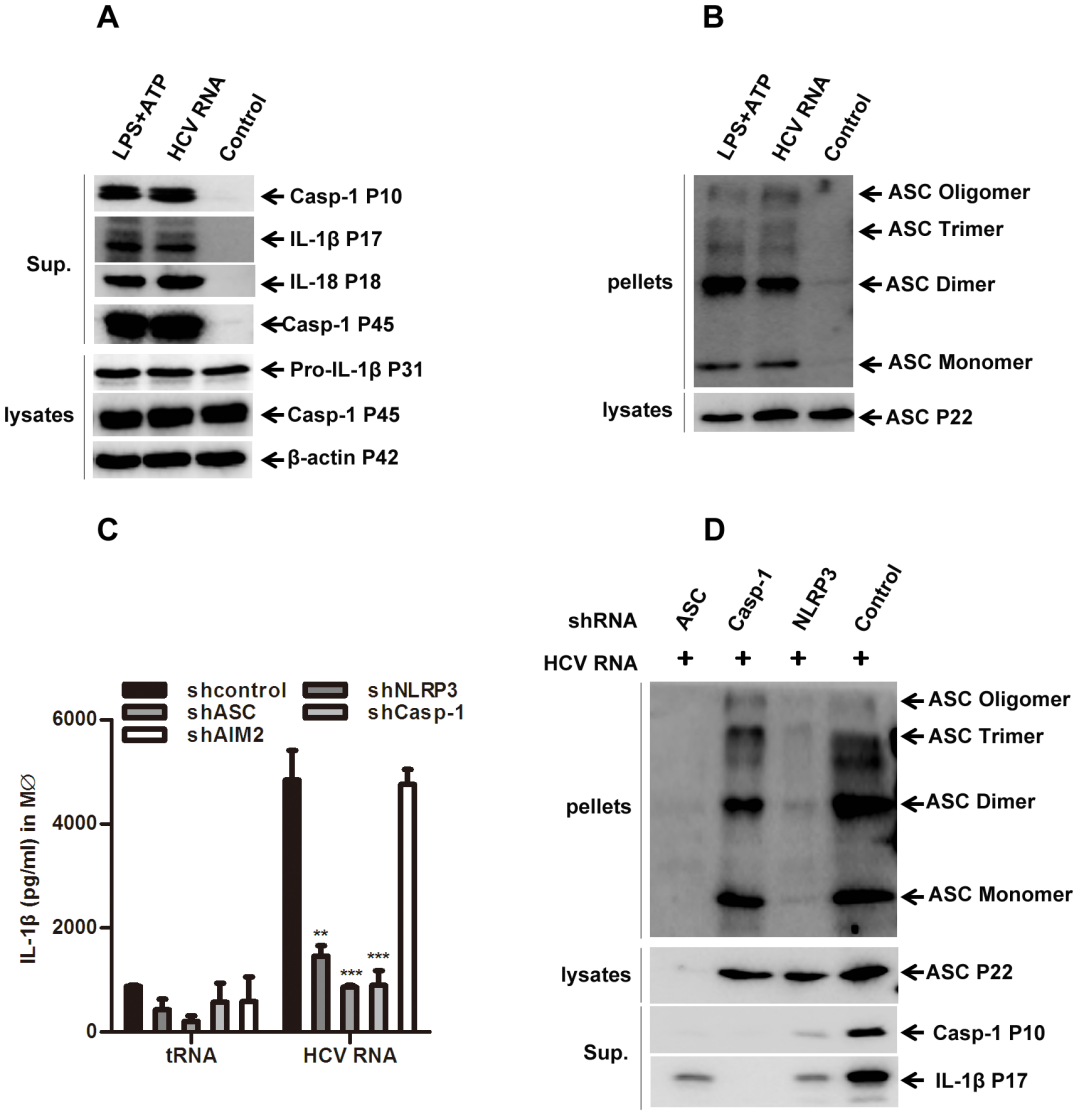
HCV RNA induces NLRP3 inflammasome activation. THP-1 derived macrophages were stimulated with HCV RNA for 6 hours, or LPS (200 ng/ml) for 6 hours followed by 5 mM ATP pulsing for 30 minutes, then the whole cell lysates were harvested for immunoblotting (A, B). C, THP-1 cells expressing specific shRNAs targeting AIM2, NLRP3, ASC, or Caspase-1 genes were differentiated into macrophages, followed by stimulation with 2 µg/ml HCV RNA for 6 hours, and then the supernatants were harvested for IL-1β ELISA. D, Cells as in (A) were stimulated with HCV RNA for 6 hours, and the supernatant and whole cell lysates were harvested for ASC specific immunoblotting. Data in C represent the means ± SD of at least three independent experiments performed with internal triplicates. A, B, D is one representative experimental result of at least three repeats, respectively. ***represents P<0.001 and **represents P<0.01 in comparison with controls during statistical analysis.

The robust IL-1β induction by HCV RNA from macrophages mentioned above implied an activation of inflammasome. The IL-1β mRNA and protein induction by HCV RNA indicated that HCV RNA could provide both signal 1 and signal 2 for inflammasome activation (Figure 3). Indeed, in LPS-primed macrophages, HCV RNA induced as much IL-1β secretion as exogenous ATP (Figure S3). As more direct evidence for inflammasome activation [39], the cleavage of caspase-1 and oligomerization of ASC in HCV RNA transfected cells was examined. We found that HCV RNA triggered the cleavage of caspase-1 and oligomerization of ASC as much as LPS+ATP in macrophages (Figure 4A–B), indicating a typical activation of inflammasome [40].

To further demonstrate the specificity of inflammasome activation by HCV RNA, we transfected the HCV RNA into macrophages derived from THP-1 cells with shRNA mediated silencing for ASC, caspase-1, NLRP3 or AIM2 genes ([41], [42] and Figure S4A). It was found that IL-1β secretion induced by HCV RNA was dependent on ASC, caspase-1 and NLRP3, but not AIM2 (Figure 4C). Similarly, ASC, caspase-1 and NLRP3 were all required for caspase-1 activation induced by HCV RNA (Figure 4D). Interestingly, the ASC oligomerization induced by HCV RNA required the presence of NLRP3 and ASC, but caspase-1 was dispensable (Figure 4D), which confirmed the recent observation that caspase-1 is dispensable for ASC oligomerization in murine cells [43]. These results thus indicated that HCV RNA activated the NLRP3 inflammasome.

### Mechanism Underlying NLRP3 Inflammasome Activation Induced by HCV RNA

**Figure 5:**
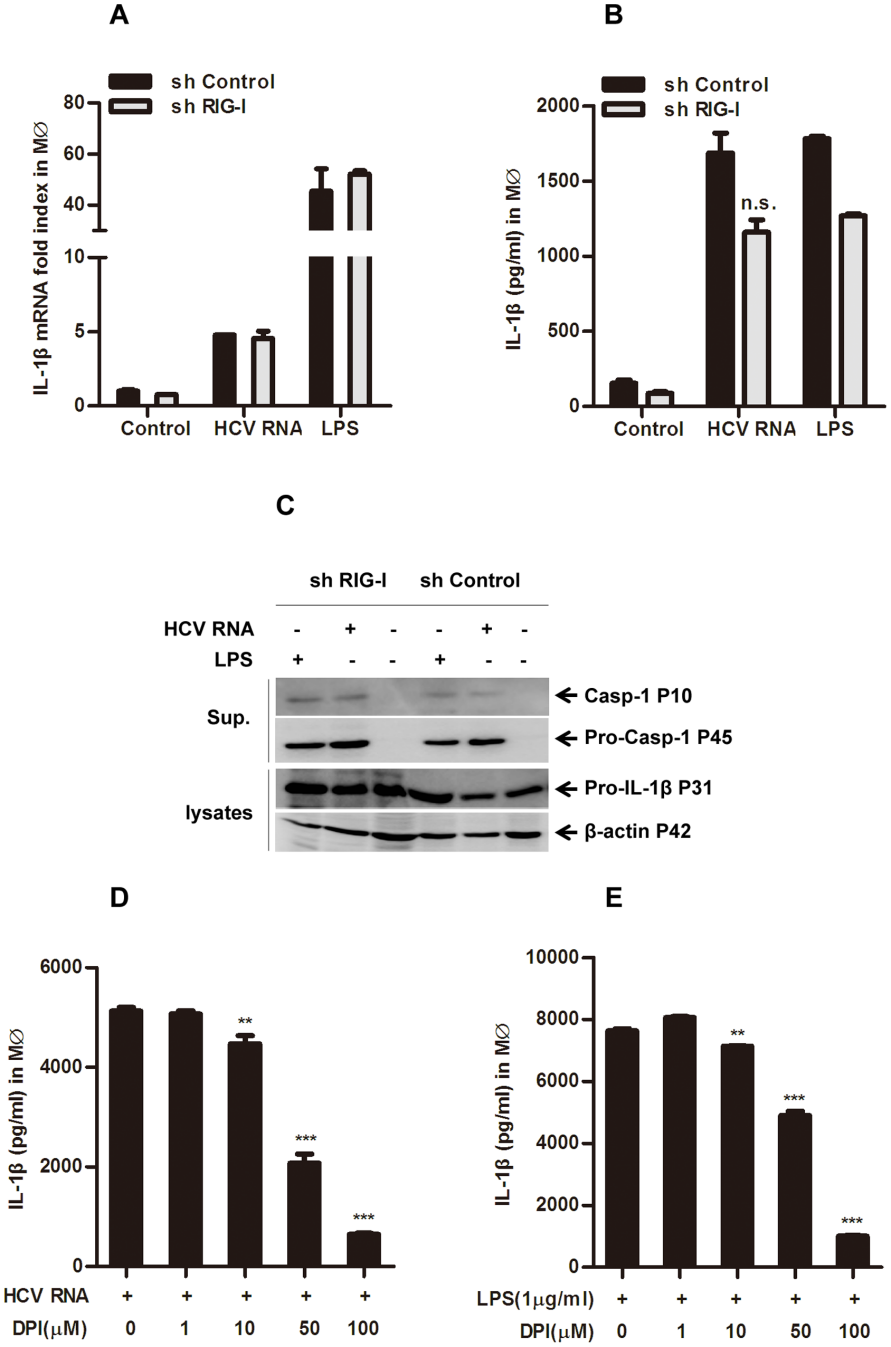
Mechanisms underlying NLRP3 inflammasome activation triggered by HCV RNA. 2 µg/ml HCV RNA was transfected in RIG-I silenced THP-1 cells, 6 hours later cells were harvested for IL1-β mRNA expression by Q-PCR (A), the supernatants were harvested for IL-1β ELISA (B). C, Cells were stimulated with HCV RNA for 6 hours, and the supernatant and whole cell lysates were harvested for immunoblotting. D–E, THP-1 derived macrophages were pretreated with ROS inhibitor DPI for half an hour, then challenged with HCV RNA (2 µg/ml) or LPS (1 µg/ml), 6 hours later the supernatants were harvested for IL-1β ELISA. Data presented are the mean ± SD of one representative figure out of three independent experiments. ***represents P<0.001, **represents P<0.01 and *represents P<0.05 in comparison with controls during statistical analysis.

More and more studies reveal that NLRP3 may not be a direct sensor for any PAMP [38], [44]. HCV RNA was reported to be recognized by RIG-I to activate IFN regulatory factor 3 and NF-kB in HCV infected Huh7 cells [5], [45]–[47]. We thus tested whether RIG-I was involved in inflammasome activation upon HCV RNA transfection. We generated shRNA targeting RIG-I in THP-1 cells and confirmed that the knock-down efficiency was significant (Figure S4B). However, when HCV RNA was transfected into such cell derived macrophages, IL-1β mRNA expression and protein secretion were not reduced in comparison with the control (Figure 5A–B). Moreover, caspase-1 cleavage was also normal in RIG-I silenced cells compared with the control upon either HCV RNA transfection or LPS stimulation (Figure 5C), while the expression of type I interferon was clearly decreased in the absence of RIG-I (Figure S5). These results indicated that in HCV RNA transfected myeloid cells, neither pro-IL-1β synthesis nor caspase-1 activation was dependent on RIG-I [25].

It is generally known that NLRP3 inflammasome-mediated cytokine release requires two signals: signal 1 activation leads to the synthesis of pro-IL-1β, pro-IL-18 and up-regulation of NLRP3 expression via NF-κB activity [48], [49]; while signal 2 can be triggered by agents or pathogens that cause potassium efflux, mitochondria damage, mtDNA release, Reactive oxygen species (ROS) production, intracellular calcium increase and cellular cyclic AMP reduction [50]–[55], which induces activation of caspase-1 and cleavage of pro-IL-1β as well as pro-IL-18. In order to explore the mechanism of NLRP3 inflammasome activation by HCV RNA, we investigated whether ROS was involved in this process. In this experiment, we pretreated THP-1 derived macrophages with ROS inhibitor diphenyliodonium (DPI) for 30 minutes, then transfected the HCV RNA into the cells before conducting the IL-1β secretion assay 6 hours later. As expected, DPI reduced HCV RNA-induced IL-1β release in a dose dependent manner (Figure 5D). LPS treatment in parallel served as a positive control (Figure 5E). These results thus reveal that HCV RNA-induced activation of the NLRP3 inflammasome was ROS-dependent.

## Discussion

In the current study, we found that HCV RNA but not whole virions activated the NLRP3 inflammasome in human myeloid cells but not in hepatocytes. Recently, many studies on inflammasome activation mediated by viruses have been reported [24], [56]–[58]. Most viruses activate the inflammasome by infecting immune cells such as macrophages and dendritic cells where inflammasome components are well expressed [56]. Although some studies indicated that NLRP3 is expressed in non-immune cells such as keratinocytes and lung epithelial cells [59], [60], its expression has not been detected in primary hepatocytes [29]. We also found that the expression level of NLRP3 in Huh7 cells was low, and was not upregulated by HCV infection. It is interesting that Burdette *et al.* found that HCV infection induced NLRP3 inflammasome activation in Huh7.5 cells [28]. However, that result could not be reproduced in our experimental system, nor in the study from Negash *et al.* [30]. Burdette *et al.* performed their study in Huh7.5 cells that are RIG-I deficient [28]. However, Negash *et al.* did not find appreciable IL-1β levels in HCV infected hepatoma cells and primary hepatocytes (PH5CH8, IHH, Huh7 and Huh7.5 cells) [30]. Although we conducted our study in Huh7 and Huh7.5.1 cells instead of Huh7.5 cells, these Huh7.5.1 cells were also RIG-I deficient hepatoma cells alike Huh7.5 cells [30]. Some unknown factor(s) in the Huh7.5 cells used by Burdette *et al*. may account for their different findings in comparison with ours and that from Negash *et al.*

Although a number of clinical discoveries provided clues that HCV infection may activate the inflammasome [8], [11]–[15], the fact that HCV cannot infect macrophages or dendritic cells, and the lack of availability of human primary hepatocytes or liver Kupffer cells made the investigation rather difficult to perform. Nonetheless, Negash *et al.* found that HCV virions activate the NLRP3 inflammasome in macrophages upon phagocytosis and HCV RNA was only responsible for pro-IL-1β synthesis, but not caspase-1 activation [30]; while in our study, HCV virions could not activate the inflammasome. Instead, we demonstrated that transfection of HCV RNA was able to activate the NLRP3 inflammasome in human myeloid cells. Our direct evidence for HCV RNA induced NLRP3 inflammasome includes the formation of the ASC pyroptosome and the cleavage of caspase-1 in macrophages. Furthermore, we found this process was dependent on NLRP3, ASC and caspase-1.

Although we demonstrated that HCV RNA was responsible for NLRP3 inflammasome activation by *in vitro* transfection, it would be interesting to investigate how this happens in physiological conditions. HCV RNA can be delivered into monocytes and/or macrophages via the following routes. Firstly, HCV RNA was reported to be delivered into human pDCs by exosomes when HCV subgenome replicon cells or JFH-1 infected Huh7 cells are co-cultured with pDCs [61], and it can be transmitted between human hepatoma Huh7.5 cells [62], which suggest that it could also be transferred into monocytes or macrophages. Secondly, non-neutralizing antibody may help macrophages engulf HCV virions to promote HCV RNA delivery and recognition *in vivo* [63], [64].

Negash and colleagues demonstrated that HCV RNA is sensed by TLR7 and induces the synthesis of pro-IL-1β through MyD88-mediated NF-κB activation, while VISA is not involved in this process. We have not investigated the possible role of TLR7 in HCV RNA induced IL-1β production, and we identified that HCV RNA induced pro-IL-1β synthesis was not RIG-I dependent. At present we could not exclude the possible involvement of TLR7 in HCV RNA triggered IL-1β production, and whether VISA plays a role during the inflammasome activation process awaits further study.

VISA was recently reported to promote NLRP3 inflammasome activation, but the role of RIG-I was not included in that work [65]. Interestingly, in our study HCV RNA did not activate caspase-1 through RIG-I. It was reported that even different strains of VSV appeared to be different in the activation of the RIG-I inflammasome [25], [56]. It could be that RIG-I inflammasome activation is specific for murine cells only upon certain virus infection.

We have not elucidated the reason why HCV virions could not induce inflammasome activation in our hands, a possible reason could be that the macrophages in our hands are not as sensitive as the cells in the study by Negash *et al*. It could also be due to some yet unknown difference between the virions produced from these two labs. As for the question of why phagocytosis of HCV virions could not activate the inflammasome while transfection of HCV RNA could, we speculate that in our system, the macrophages require a larger amount of HCV RNA for inflammasome activation, which can only be fulfilled through transfection. Phagocytosis of virions might not provide sufficient amount of HCV RNA for activation. However, this recognition of HCV RNA may happen in physiologic conditions through exosome-mediated delivery or non-neutralizing antibody-mediated engulfment.

Interestingly, we demonstrated that only certain portions of the HCV RNA, which includes the 3′UTR, could activate the NLRP3 inflammasome efficiently. The other portions tested (1–807 bp, 2406–3256 bp, 5626–6437 bp) were not able to do so. However, the 3′UTR was still not as potent as the full length HCV genomic RNA in activating the inflammasome, indicating how other motifs may also involved in the activation process. Negash *et al*. speculated that transient production of p7 and other HCV proteins might provide stimuli (such as signal 2) for inflammasome activation [30], and during the revision of our study, Shrivastava *et al.* published their observation that HCV P7 RNA induced IL-1β secretion in macrophages in a way slightly weaker than HCV genomic RNA [26]. It would be interesting to test whether there is any synergistic effect when 3′UTR and P7 RNA are co-transfected.

We verified that ROS was involved in HCV RNA-induced inflammasome activation, and HCV RNA was able to activate both signal 1 and signal 2 in human myeloid cells as many other PAMPs and microbes do [41]. We have not studied whether other mechanisms such as potassium efflux, calcium influx and mitochondrial mtDNA release are related to HCV RNA-induced NLRP3 inflammasome activation [50]–[55], which deserves further investigation.

In summary, we have identified that HCV RNA but not virions could activate the NLRP3 inflammasome. RIG-I was not required for the activation, while ROS production was involved in this process. Our study thus provided a novel route of inflammation observed in HCV infected patients.

## Supporting Information

Figure S1 **HCV infection does not induce IL-1β secretion from Huh7.5.1 cells.** Huh7.5.1 cells were incubated with HCV virions (MOI = 1) for 4 days, then supernatants were harvested for IL-1β ELISA. LPS treated THP-1 mococytic cells was set as positive control. Data are mean ± SD of one representative out of three independent experiments.
(TIF)

Figure S2 **HCV infection does not induce IL-1β production from THP-1 derived macrophages.** THP-1 cells were differentiated to macrophages by treatment with 40 nM of PMA overnight at 37°C as described by Negash et al [30]. These macrophages were incubated with purified HCV virions with indicated MOI for 12 hours and the supernatants were harvested for IL-1β ELISA. Data presented are mean ± SD of one representative out of three independent experiments.
(TIF)

Figure S3 **HCV RNA induces IL-1β from LPS-primed macrophages.** THP-1 derived macrophages primed or non-primed with 100 ng/ml LPS for 6 hours were stimulated with 1 ug/ml LPS or transfected 2 µg/ml HCV RNA for 6 hours or 5 mM ATP for half an hour and the supernatants were harvested for IL-1β ELISA. Data presented are mean ± SD of one representative out of three independent experiments.
(TIF)

Figure S4 **The knock-down efficiency of AIM2 and RIG-I in respective THP-1 cells.** Q-PCR was applied to monitor the expression of AIM2 or RIG-I in shRNA transfected THP-1 cells,AIM2-1 and RIG-I-3 were used for experiments in our study.
(TIF)

Figure S5 **IFN-β induction by HCV RNA is dependent on RIG-I.** 2 µg/ml HCV RNA was transfected into macrophages derived from THP-1 cells silenced for RIG-I, 6 hours later the cells were harvested for IFN-β mRNA expression by Q-PCR. The values represent mean value ± SD of three independent experiments. **represents P<0.01 in comparison with control in statistic analysis.
(TIF)
