# Supplementary figures and images for: HCV Genomic RNA Activates the NLRP3 Inflammasome in Human Myeloid Cells
Source: PLoS One. 2014 Jan 6;9(1):e84953. doi: 10.1371/journal.pone.0084953 (PMC3882267; doi:10.1371/journal.pone.0084953)

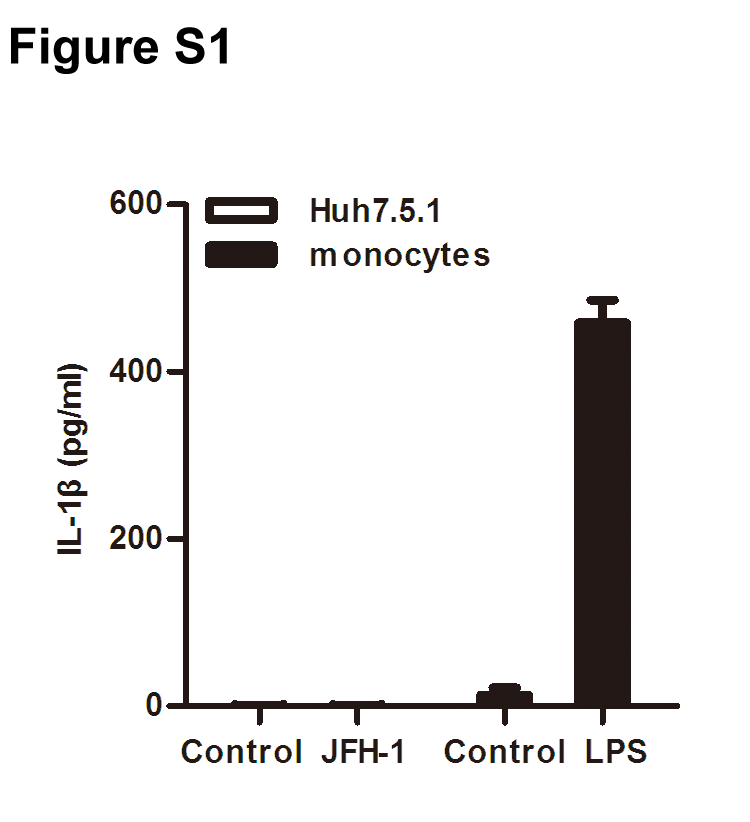

Supplement: Figure S1 — HCV infection does not induce IL-1β secretion from Huh7.5.1 cells. Huh7.5.1 cells were incubated with HCV virions (MOI = 1) for 4 days, then supernatants were harvested for IL-1β ELISA. LPS treated THP-1 mococytic cells was set as positive control. Data are mean ± SD of one representative out of three independent experiments. (TIF) [file pone.0084953.s001.tif]

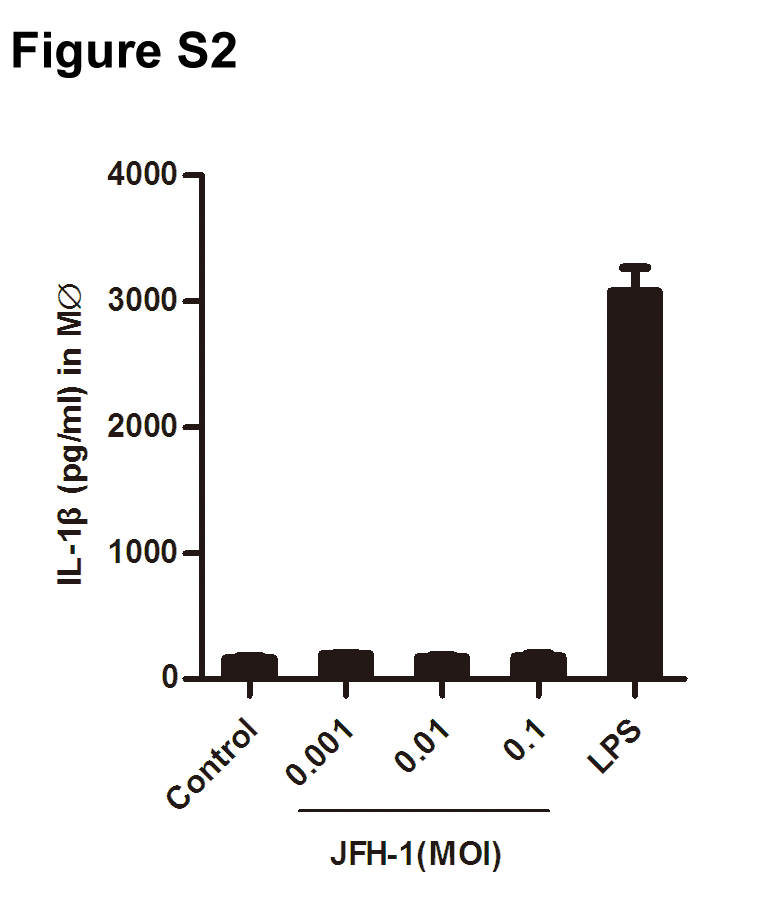

Supplement: Figure S2 — HCV infection does not induce IL-1β production from THP-1 derived macrophages. THP-1 cells were differentiated to macrophages by treatment with 40 nM of PMA overnight at 37°C as described by Negash et al [30]. These macrophages were incubated with purified HCV virions with indicated MOI for 12 hours and the supernatants were harvested for IL-1β ELISA. Data presented are mean ± SD of one representative out of three independent experiments. (TIF) [file pone.0084953.s002.tif]

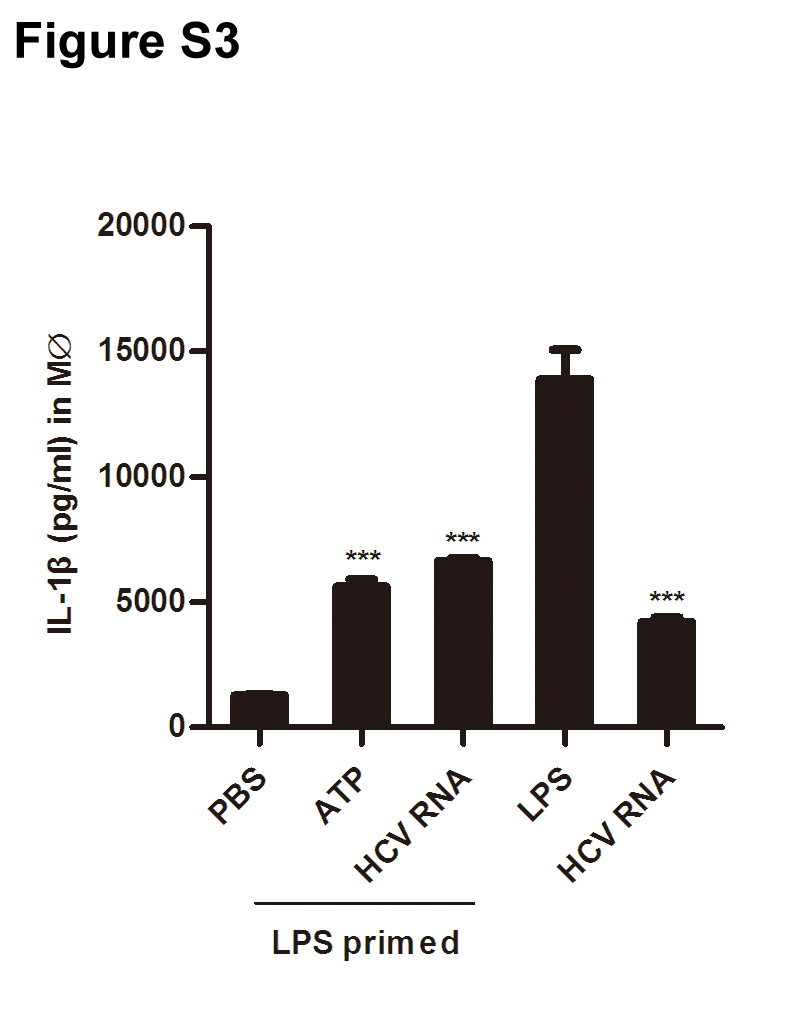

Supplement: Figure S3 — HCV RNA induces IL-1β from LPS-primed macrophages. THP-1 derived macrophages primed or non-primed with 100 ng/ml LPS for 6 hours were stimulated with 1 ug/ml LPS or transfected 2 µg/ml HCV RNA for 6 hours or 5 mM ATP for half an hour and the supernatants were harvested for IL-1β ELISA. Data presented are mean ± SD of one representative out of three independent experiments. (TIF) [file pone.0084953.s003.tif]

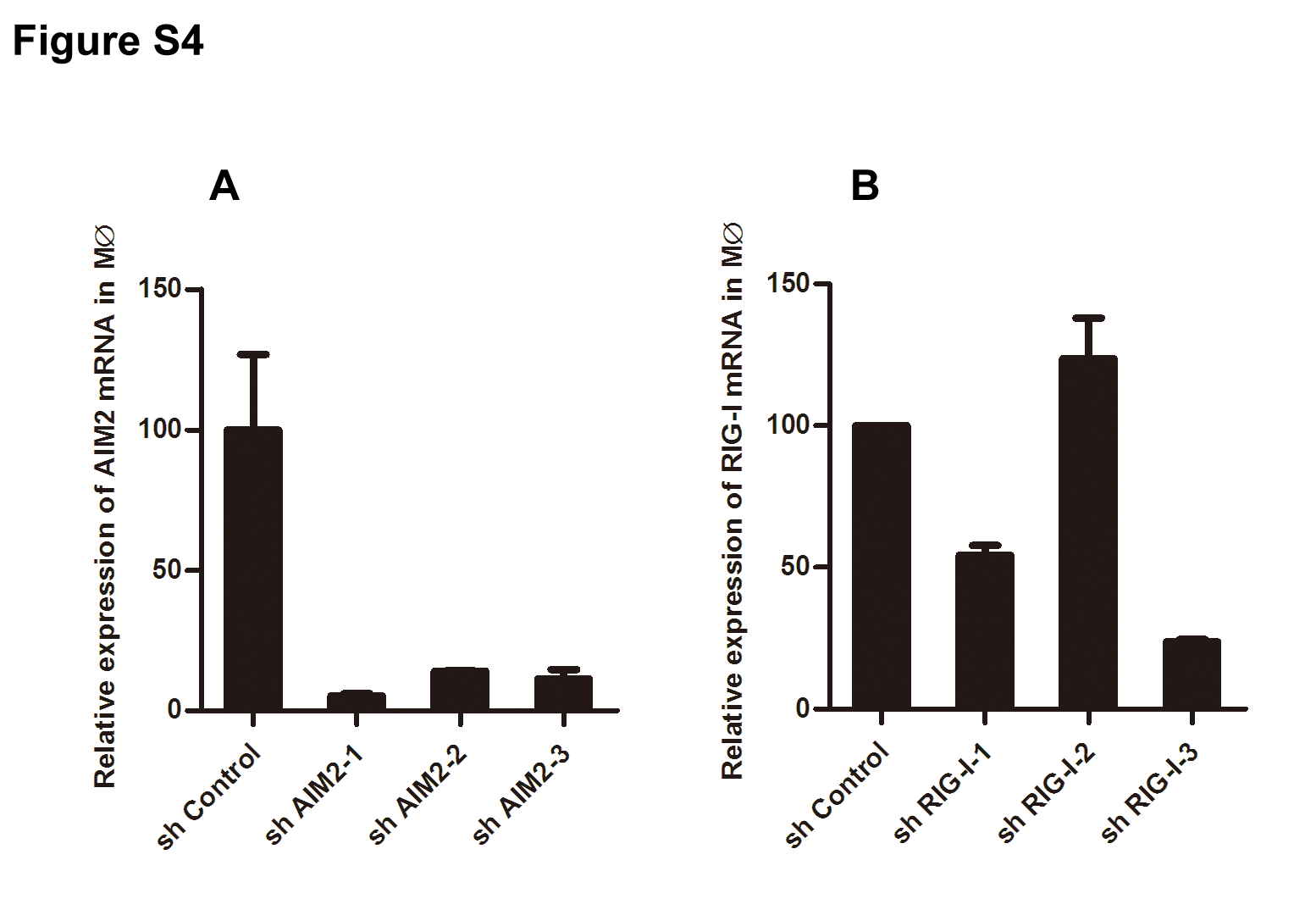

Supplement: Figure S4 — The knock-down efficiency of AIM2 and RIG-I in respective THP-1 cells. Q-PCR was applied to monitor the expression of AIM2 or RIG-I in shRNA transfected THP-1 cells,AIM2-1 and RIG-I-3 were used for experiments in our study. (TIF) [file pone.0084953.s004.tif]

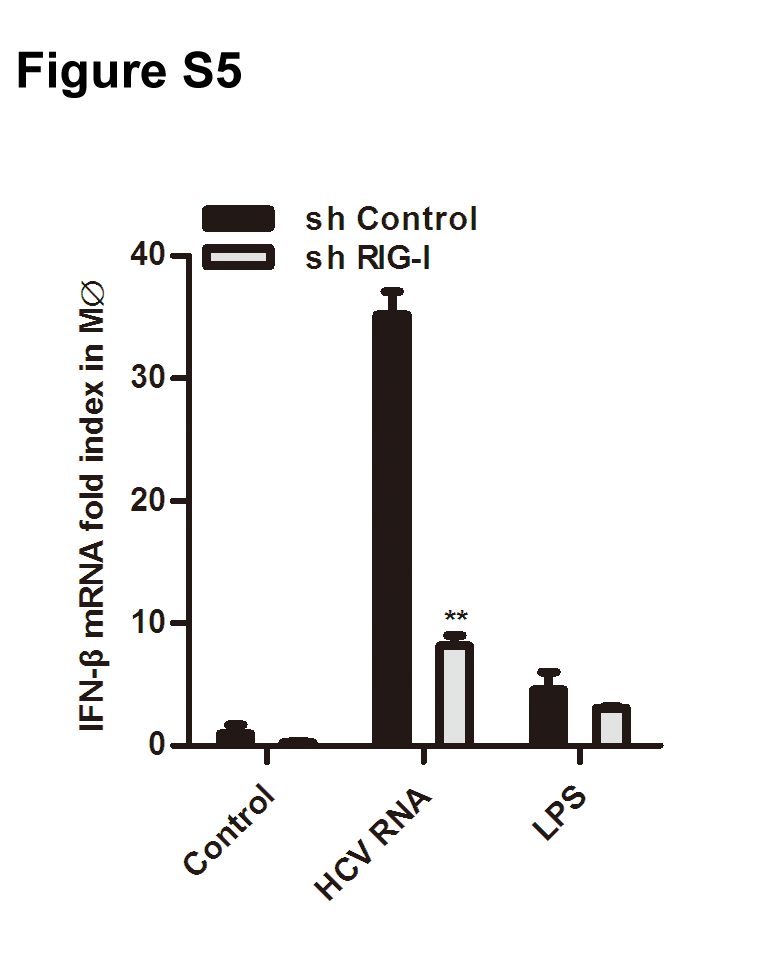

Supplement: Figure S5 — IFN-β induction by HCV RNA is dependent on RIG-I. 2 µg/ml HCV RNA was transfected into macrophages derived from THP-1 cells silenced for RIG-I, 6 hours later the cells were harvested for IFN-β mRNA expression by Q-PCR. The values represent mean value ± SD of three independent experiments. **represents P<0.01 in comparison with control in statistic analysis. (TIF) [file pone.0084953.s005.tif]
